# Feasibility and Short-Term Outcomes of One-Step and Two-Step Sleeve Gastrectomy as Revision Procedures for Failed Adjustable Gastric Banding Compared With Those After Primary Sleeve Gastrectomy

**DOI:** 10.3389/fsurg.2021.752319

**Published:** 2021-09-22

**Authors:** Omar Thaher, Jamal Driouch, Martin Hukauf, Ferdinand Köckerling, Christine Stroh

**Affiliations:** ^1^Department of Surgery, Marien Hospital Herne, Ruhr-Universität Bochum, Herne, Germany; ^2^StatConsult Society for Clinical and Health Services Research GmbH, Magdeburg, Germany; ^3^Department of Surgery and Center for Minimally Invasive Surgery, Academic Teaching Hospital of Charité Medical School, Vivantes Hospital, Berlin, Germany; ^4^Department of General, Abdominal and Pediatric Surgery, Municipal Hospital, Gera, Germany

**Keywords:** sleeve gastrectomy, gastric banding, one-step procedure, two-step procedure, perioperative complications

## Abstract

**Background:** The practice of bariatric surgery was studied using the German Bariatric Surgery Registry (GBSR). The focus of the study was to evaluate whether revision surgery One-Step (OS) or Two-Step (TS) sleeve gastrectomy (SG) has a large benefit in terms of perioperative risk in patients after failed Adjustable Gastric Banding (AGB).

**Methods:** The data collection includes patients who underwent One-Step SG (OS-SG) or Two-Step SG (TS-SG) as revision surgery after AGB and primary SG (P-SG) between 2005 and 2019. Outcome criteria were perioperative complications, comorbidities, 30-day mortality, and operating time.

**Results:** The study analyzed data from 27,346 patients after P-SG, 320 after OS-SG, and 168 after TS-SG. Regarding the intraoperative complication, there was a significant difference in favor of P-SG and TS-SG compared to OS-SG (*p* < 0.001). The incidence of pulmonary complications was significantly higher in the OS-SG (*p* < 0.001). There was also a significant difference in occurrence of staple line stenosis in favor of TS-SG (*p* = 0.005) and the occurrence of sepsis (*p* = 0.008). The mean operating time was statistically longer in the TS-SG group than in the OS-SG group (*p* < 0.001). The 30-day mortality was not significantly different between the three groups (*p* = 0.727).

**Conclusion:** In general, our study shows that converting a gastric band to a SG is safe and feasible. However, lower complications were obtained with TS-SG compared to OS-SG. Despite acceptable complication and mortality rates of both procedures, we cannot recommend any surgical method as a standard procedure. Proper patient selection is crucial to avoid possible adverse effects.

## Introduction

Extreme obesity is a severe clinical problem in the Western world (1). In recent decades, bariatric surgery, regardless of the type of surgical procedure used, has shown great success in changing body mass index (BMI) compared to the results of non-surgical procedures (2). Bariatric surgery is recommended for people who have not achieved permanent %total weight loss (%TWL) with non-surgical methods (3). A well-known bariatric surgical procedure for treating severe obesity is adjustable gastric banding (AGB) (4). Unfortunately, AGB requires revision surgery in 20–60% of cases due to band slippage, chronic esophagitis, erosion, pouch dilation, infection, discomfort, and complications (i.e., vomiting, infection or positioning problems, pain after eating, or difficulty swallowing) or failure with significant %TWL (5–7). Here, surgeons should use other surgical procedures to achieve tremendous %TWL and effective complication management. One of the most commonly used surgical procedures is Sleeve Gastrectomy (SG). Several clinical studies investigated the effect of SG on BMI change and complication management as revision surgery after failed gastric banding (8–10). Our study aims to show the safety of One-Step vs. Two-Step revision surgery from AGB to SG and compare the results with those after P-SG. The long-term outcomes, such as %TWL and change in BMI, were not investigated in our study.

## Materials and Methods

This retrospective study with prospectively collected data analyzed data from patients who underwent revisional SG surgery (R-SG) after AGB failure and primary sleeve gastrectomy between 2005 and 2019. Processed data from the export of the Quality Assurance Study for Surgical Therapy of Obesity of the German Bariatric Surgery Registry (GBSR) of the Institute for Quality Assurance in Surgical Medicine of the Otto-von-Guericke University Magdeburg were used. Only interventions that were validated as plausible at the time of data export were included in our analysis. Plausibility checks were performed when preparing obesity data for annual reports. Data included demographic and medical aspects such as age, sex, comorbidities, BMI (kg/m^2^), 30-day mortality, operating time (from surgery start time to surgery end time in minutes), and peri- and post-operative complications.

All analyses were performed by StatConsult GmbH using SAS 9.4 software (SAS Institute Inc., Cary, NC, USA). As this was an exploratory analysis, the overall significance level of 5% was deliberately used, i.e., no correction for multiple testing is applied, and any *p* ≤ 0.05 corresponds to a significant result.

In our study, data from 27,834 patients were analyzed. We focused only on the results of SGs as the primary procedure and revision procedures after failed AGB. For revision SG, we did not discuss and analyze the reasons for band removal and reoperation. The distributions of (quasi-) continuous variables, the mean and standard deviation (STD), were reported in the results tables. For categorical variables, relative (%) frequencies were presented in contingency tables. The chi-square test was used for unadjusted analyses of procedures (P vs. OS vs. TS). Analysis of variance (ANOVA) was used for continuous variables. Analysis of non-normally distributed data (operative time) was performed with log-transformed values.

Our analysis included various medical aspects, such as intraoperative and post-operative complication rates, 30-day mortality, comorbidities, and operative time. In addition, the specific post-operative complications (SPC) such as sepsis, abscess formation, bleeding requiring transfusion, bleeding requiring surgery, and staple line leak were studied. Intraoperative complications such as splenic, biliary, hepatic, and vascular injuries, pneumothorax, gastric perforation, and intraoperative bleeding were analyzed. In addition to P-SG, our study compared the perioperative outcomes of revision surgery OS-SG vs. TS-SG after AGB. In the first step, we compared the outcome of patients after revision surgery (*n* = 488) with those who underwent P-SG. In the second step, we compared the outcome of patients after OS-SG with those who underwent TS-SG. All patients with the TS-SG procedure underwent primary removal of the gastric banding, and a SG was performed later. The mean operative time for the TS-SG included the time for band removal and the time for the SG. The interval for the Two-Step procedure between band removal and SG was set at ~6 weeks to 6 months to ensure favorable tissue conditions. Since this is a registry data collection, we cannot describe the surgical steps for band removal and revision surgery. It depends on the surgeons and their expertise which method they use in the procedure. In our analysis, the type of surgery (laparoscopic vs. open approach) was not considered.

## Results

**Figure 1:**
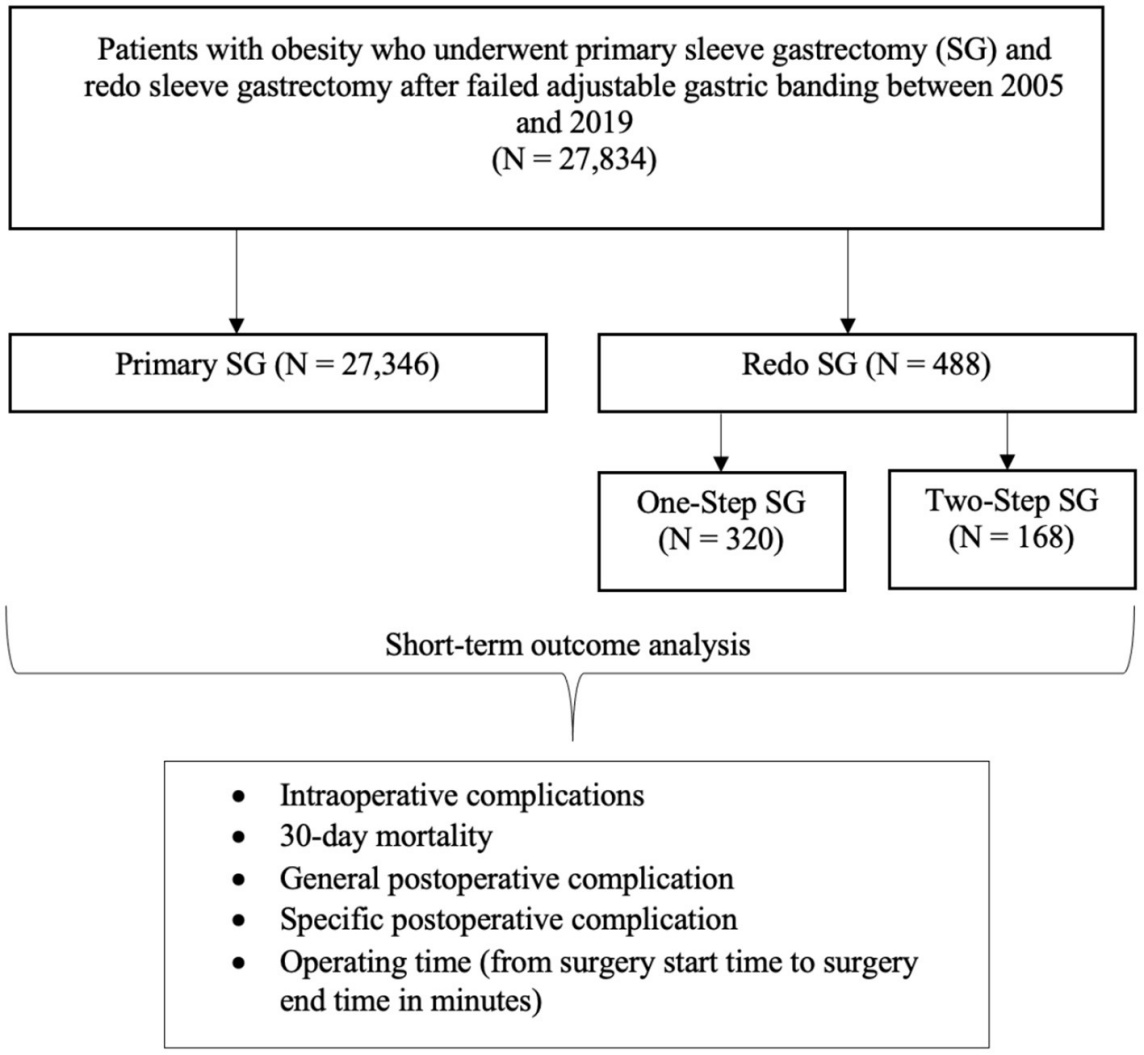
Flowchart of patient inclusion.

We analyzed data from 27,834 patients from 2005 to 2019; 27,346 patients underwent P-SG, 320 patients OS-SG, and 168 patients TS-SG (Figure 1; Table 1).

**Table 1:** Distribution of patients according to demographic variables and surgical method.

		**P-SG**	**OS-SG**	**TS-SG**	***p*-value**
Age (y)	Mean value ± STD	43.5 ± 11.6	45.4 ± 9.8	46.2 ± 8.9	<0.001
BMI Kg/m^2^	Mean value ± STD	51.3 ± 9.2	45.3 ± 11.2	46.8 ± 9.6	<0.001
Gender (m/f)	%	34.3/65.7	25.9/74.1	29.8/70.2	0.004
	(*n*)	9,378/17,968	83/237	50/118	
**Distribution of surgical method**					
**Method**		**Total**			
			**(** ***n*** **)**	**%**	
AGB to SG		OS-SG	320	66	
		TS-SG	168	34	
		Total	488		
P-SG			27,346		
Total			27,834		

### Descriptive Statistics and Unadjusted Analyses

The continuous parameters of the perioperative course for all patients who underwent P-SG, OS-SG, and TS-SG surgery are shown in Table 1. There was a significant difference in mean BMI and age between the three groups. Thus, P-SG patients had significantly higher BMI (51.3 ± 9.2 kg/m^2^ P-SG vs. 45.3 ± 11.2 OS-SG vs. 46.8 ± 9.6 TS-SG; *p* < 0.001). TS-SG patients were significantly older than P-SG but almost identical to OS-SG patients (43.5 ± 11.6 years P-SG vs. 45.4 ± 9.82 OS-SG vs. 46.2 ± 8.9 TS-SG; *p* < 0.001). In addition, we analyzed the percentage distribution of male patients who underwent bariatric surgery compared to female patients. It was found that the number of female patients accounted for a larger proportion than the number of male patients [34.3/65.7% (m/f) P-SG vs. 25.9/74.1% OS-SG vs. 29.8/70.2% TS-SG; *p* = 0.004].

The percentage distribution of comorbidities was significantly higher in P-SG than in OS- and TS-SG (90.7% in P-SG group vs. 78.1% in OS-SG and 79.8% in TS-SG group; *p* < 0.001). Thirty-four percentage of patients in the P-SG group had type 2 diabetes mellitus (T2DM) vs. 25.3% in the OS- and 27.3% in the TS-SG group (*p* = 0.002). The rate of arterial hypertension was significantly higher in the P-SG group (62.5% P-SG vs. 50.3% OS-SG vs. 57.1 TS-SG; *p* < 0.001). Sleep apnea was diagnosed significantly more frequently in the P-SG group (27.6%) than in the OS- and TS-SG groups (18.4 and 18.5%, respectively; *p* < 0.001). The ASA classification showed a significant difference between the three groups. In comparison, ASA II was higher in OS-SG (41% in P-SG vs. 46.6% in OS-SG and 44% in TS-SG; *p* < 0.001), but ASA III was significantly higher in P-SG (52.6%) compared to 39.7% in OS-SG and 48.2% in TS-SG (*p* < 0.001). A significant difference was also found in the three groups regarding pulmonary comorbidities (20.4% P-SG vs. 11.6% OS-SG vs. 14.9% TS-SG; *p* < 0.001). The rate of reflux symptoms was significantly higher in the OS-SG group (12.2% in the P-SG group, 16.6% in the OS-SG group, and 8.3% in the TS-SG; *p* = 0.019). The distribution of other comorbidities such as degenerative spine disease (DSD), degenerative skeletal disease, and gonarthrosis was also significantly different among the three groups. While DSD (35.1% in P-SG, 34.1% in OS-SG and 25.6% in TS-SG; *p* = 0.034) and gonarthrosis (23.2% in P-SG, 15.3% in OS-SG and 16.1% in TS-SG; *p* < 0.001) were higher in P-SG, degenerative skeletal disease was significantly higher in OS-SG (48.8%) than in P-SG (46%) and in TS-SG (31.5%; *p* < 0.001). There were no significant differences between the three groups in the incidence of pulmonary embolism (*p* = 0.370), other cardiovascular diseases (*p* = 0.344), varicose veins (*p* = 0.819), non-alcoholic steatohepatitis (NASH) (*p* = 0.327), and nicotine abuse (*p* = 0.527). A summary of the comorbidities is shown in Table 2.

**Table 2:** Distribution of patients according to comorbidities.

		**P-SG**		**OS-SG**		**TS-SG**		***p*-value**
		***n***	**%**	***n***	**%**	***n***	**%**	
ASA	ASA I	1,131	4.1	31	9.7	9	5.4	<0.001
	ASA II	11,193	41.0	149	46.6	74	44.0	
	ASA III	14,345	52.6	127	39.7	81	48.2	
	ASA IV	628	2.3	13	4.1	4	2.4	
Comorbidities	Yes	24,811	90.7	250	78.1	134	79.8	<0.001
	No	2,535	9.3	70	21.9	34	20.2	
Diabetes (total) T2DM	Yes	8,656	34.0	75	25.3	42	27.3	0.002
	No	16,832	66.0	221	74.7	112	72.7	
T2DM (IDDM)	Yes	2,655	10.4	24	8.1	9	5.8	0.079
	No	22,833	89.6	272	91.9	145	94.2	
T2DM (NIDDM)	Yes	4,628	18.2	44	14.9	25	16.2	0.286
	No	20,860	81.8	252	85.1	129	83.8	
T2DM (dietary)	Yes	1,373	5.4	7	2.4	8	5.2	0.071
	No	24,115	94.6	289	97.6	146	94.8	
Arterial hypertension	Yes	17,086	62.5	161	50.3	96	57.1	<0.001
	No	10,260	37.5	159	49.7	72	42.9	
Pulmonary	Yes	5,579	20.4	37	11.6	25	14.9	<0.001
	No	21,767	79.6	283	88.4	143	85.1	
Pulmonary embolism	Yes	316	1.2	1	0.3	2	1.2	0.370
	No	27,030	98.8	319	99.7	166	98.8	
Other cardiac and vascular diseases	Yes	3,020	11.0	30	9.4	14	8.3	0.344
	No	24,326	89.0	290	90.6	154	91.7	
Reflux	Yes	3,343	12.2	53	16.6	14	8.3	0.019
	No	24,003	87.8	267	83.4	154	91.7	
Degenerative Spine disease	Yes	9,595	35.1	109	34.1	43	25.6	0.034
	No	17,751	64.9	211	65.9	125	74.4	
Varicoses	Yes	1,700	6.2	18	5.6	9	5.4	0.819
	No	25,646	93.8	302	94.4	159	94.6	
Non-alcoholic steatohepatitis (NASH)	Yes	1,524	7.1	10	5.6	5	4.1	0.327
	No	20,042	92.9	168	94.4	118	95.9	
Degenerative skeletal disease	Yes	12592	46.0	156	48.8	53	31.5	<0.001
	No	14,754	54.0	164	51.3	115	68.5	
Nicotine abuse	Yes	2,814	10.3	27	8.4	16	9.5	0.527
	No	24,532	89.7	293	91.6	152	90.5	
Gonarthrosis	Yes	6,334	23.2	49	15.3	27	16.1	<0.001
	No	21,012	76.8	271	84.7	141	83.9	
Sleep apnea	Yes	7,543	27.6	59	18.4	31	18.5	<0.001
	No	19,803	72.4	261	81.6	137	81.5	

### Operation Data and Time

Since over 95% of the procedures were performed laparoscopically, the type of surgery (laparoscopic vs. open vs. conversion) was not considered in the analysis (Table 3). However, OS-SG had a significantly higher documented conversion rate than P-SG and TS-SG (0.4% for P-SG vs. 2.2% for OS-SG vs. 0.6% for TS-SG; *p* < 0.001). The mean operative time was statistically longer in TS-SG patients than in OS-SG patients (162.3 vs. 113.8 min; *p* < 0.001). The mean surgery time of TS-SG included both procedures (band removal in the first step and SG in the second step). Compared to P-SG (74.8 min), the operating time for OS - and TS-SG was significantly longer (*p* < 0.001).

**Table 3:** Operative data.

**Type of surgery**							
	**P-SG**		**OS-SG**		**TS-SG**		***p*-value**
	***n***	**%**	***n***	**%**	***n***	**%**	
Laparotomy	194	0.7	6	1.9	2	1.2	<0.001
Laparoscopy	26,962	98.9	306	95.9	164	98.2	
Conversion	118	0.4	7	2.2	1	0.6	
Operation time (min)* [mean (range)]	74.8 (73.3; 76.4)		113.8 (112.3; 115.4)		162.3 (160.7; 163.8)		<0.001

### Intraoperative and Post-operative Complication Rates and 30-Day Mortality

A total of 407 intraoperative complications were documented. 390 (1.4%) of these complications occurred in the P-SG, 12 (3.8%) in the OS-SG, and 5 (3%) in the TS-SG. There was a significant difference in favor of P-SG and TS-SG compared to OS-SG (*p* < 0.001). There were significant disadvantages between the three groups regarding gastric perforation in favor of TS-SG (<0.1% for the P-SG vs. 0.9% for OS-SG and 0% for TS-SG; *p* < 0.001). The splenic injury was significantly higher in TS-SG (0.3% for P-SG vs. 0.6% for OS-SG and 1.8% for TS-SG; *p* = 0.003). There were no significant disadvantages between the three groups regarding other complications, with an overall *p*-value of 0.05. Regarding 30-day mortality, no significant difference was found between the three groups (0.2% P- SG vs. 0.3% OS-SG vs. 0% TS-SG; *p* = 0.727). The details of complications are summarized in Table 4.

**Table 4:** Intraoperative complications.

		**P-SG**		**OS-SG**		**TS-SG**		***p*-value**
		***n***	**%**	***n***	**%**	***n***	**%**	
Intraoperative complication (total)	Yes	390	1.4	12	3.8	5	3.0	<0.001
	No	26,956	98.6	308	96.3	163	97.0	
Injury of splenic	Yes	87	0.3	2	0.6	3	1.8	0.003
	No	27,259	99.7	318	99.4	165	98.2	
Injury of liver	Yes	21	<0.1	1	0.3	0	0	0.307
	No	27,325	>99.9	319	99.7	168	100	
Pneumothorax	Yes	7	<0.1	0	0	0	0	0.939
	No	27,339	>99.9	320	100	168	100	
Perforation of the stomach	Yes	16	<0.1	3	0.9	0	0	<0.001
	No	27,330	>99.9	317	99.1	168	100	
Bile duct injury	Yes	3	<0.1	0	0	0	0	0.974
	No	27,343	>99.9	320	100	168	100	
Vascular injury	Yes	23	<0.1	1	0.3	0	0	0.357
	No	27,323	>99.9	319	99.7	168	100	
Bleeding	Yes	21	<0.1	0	0	0	0	0.829
	No	27,325	>99.9	320	100	168	100	
30-day mortality	Yes	48	0.2	1	0.3	0	0	0.727
	No	27,275	99.8	318	99.7	168	100	

The total general post-operative complication rate was not significantly different between the three groups (*p* = 0.267). However, regarding individual complications, the incidence of pulmonary complications was significantly higher in the OS-SG (1% P-SG vs. 3.1% OS-SG vs. 0% TS-SG; *p* < 0.001). With a *p*-value above 0.05, there was no significant difference between the three groups in the incidence of urinary tract infections (*p* = 0.267), cardiac and renal complications (*p* = 0.6 and *p* = 0.792), fever (*p* = 0.051), thrombosis (*p* = 0.525) and other complications (*p* = 0.496). A summary of the general post-operative complications is shown in Table 5.

**Table 5:** General and special post-operative complications.

		**P-SG**		**OS-SG**		**TS-SG**		
		***n***	**%**	***n***	**%**	***n***	**%**	***p*-value**
**General post-operative complication**								
Total	Yes	1,281	4.7	21	6.6	9	5.4	0.267
	No	26,065	95.3	299	93.4	159	94.6	
Urinary tract infection	Yes	223	0.8	0	0	2	1.2	0.231
	No	27,123	99.2	320	100	166	98.8	
Cardiac complication	Yes	116	0.4	2	0.6	0	0	0.600
	No	27,230	99.6	318	99.4	168	100	
Renal complication	Yes	83	0.3	1	0.3	1	0.6	0.792
	No	27,263	99.7	319	99.7	167	99.4	
Pulmonary complication	Yes	266	1.0	10	3.1	0	0	<0.001
	No	27,080	99.0	310	96.9	168	100	
Fever	Yes	266	1.0	6	1.9	4	2.4	0.051
	No	27,080	99.0	314	98.1	164	97.6	
Thrombosis	Yes	31	0.1	1	0.3	0	0	0.525
	No	27,315	99.9	319	99.7	168	100	
Other	Yes	660	2.4	11	3.4	4	2.4	0.496
	No	26,686	97.6	309	96.6	164	97.6	
**Special post-operative complication**								
Total	Yes	1,046	3.8	20	6.3	5	3.0	0.068
	No	26,300	96.2	300	93.8	163	97.0	
Bleeding requiring transfusion	Yes	213	0.8	4	1.3	2	1.2	0.535
	No	27,133	99.2	316	98.8	166	98.8	
Bleeding requiring surgery	Yes	341	1.2	3	0.9	0	0	0.307
	No	27,005	98.8	317	99.1	168	100	
Staple line leak	Yes	315	1.2	8	2.5	1	0.6	0.065
	No	27,031	98.8	312	97.5	167	99.4	
Stenosis	Yes	23	<0.1	2	0.6	0	0	0.005
	No	27,323	>99.9	318	99.4	168	100	
Ileus	Yes	9	<0.1	0	0	0	0	0.923
	No	27,337	>99.9	320	100	168	100	
Abscess formation	Yes	179	0.7	4	1.3	1	0.6	0.423
	No	27,167	99.3	316	98.8	167	99.4	
Sepsis	Yes	121	0.4	5	1.6	0	0	0.008
	No	27,225	99.6	315	98.4	168	100	
Peritonitis	Yes	137	0.5	3	0.9	0	0	0.129
	No	27,209	99.5	317	99.1	168	100	
Wound infection	Yes	137	0.5	3	0.9	0	0	0.343
	No	27,209	99.5	317	99.1	168	100	

The total rate of SPC was not significant in the three groups (3.8% in P-SG vs. 6.3% in OS-SG and 3% in TS-SG; *p* = 0.068). There was a significant difference in staple line stenosis (<0.1% in P-SG vs. 0.6% in OS-SG, and 0% in TS-SG; *p* = 0.005) and the occurrence of sepsis (0.4% in P-SG vs. 1.6% in OS-SG, and 0% in TS-SG; *p* = 0.008). No significant difference was found between the three groups regarding the incidence of abscess formation (*p* = 0.423), bleeding rate with a transfusion or with reoperation (*p* = 0.535 and *p* = 0.307), peritonitis (*p* = 0.129), staple line leak (*p* = 0.065), and wound infection (*p* = 0.343). Table 5 summarizes the general and specific post-operative complications.

## Discussion

Since January 1, 2005, primary and repeat bariatric procedures have been recorded as part of a Quality Assurance Study for Surgical Therapy of Obesity by the Institute for Quality Assurance Surgical Medicine at Otto-von-Guericke University Magdeburg to improve the quality of care. Comparisons were made between patients with revisional SG as One- or Two-Step procedures or primary SG.

It should be noted that (significant) results must always be discussed in context and especially considering their relevance. Significant differences in the number of patients can be achieved with small cases, especially as this is considered exploratively without adjusting for multiple testing levels. On the other hand, effects are partly descriptively visible but statistically not provable due to the small number of cases. As this is data from a registered study, it must also be taken into account that the cleanliness of the data cannot be assumed. Furthermore, only the existing data are evaluated. A bias due to incorrect values can therefore not be ruled out.

According to the results of various literature, there are many advantages for the use of adjustable gastric banding (AGB) for obesity treatment (11, 12). One study reported a permanent change in BMI with 47% EWL maintained for up to 15 years after AGB (13). However, due to the experience of AGB and its disadvantages, the rate of surgery has decreased in Europe and worldwide and another type of surgery should eliminate the complication or achieve a significant %TWL in patients with obesity (14–16). Therefore, due to the high risks of revision surgery, reoperation after failed bariatric surgery must be performed in consultation with the patient (17). Regarding revision surgery after AGB, there is no strict consensus on the optimal conversion method for patients who require revision surgery after the AGB procedure.

Some options include band repositioning or conversion to other surgical procedures. Since SG is an effective bariatric procedure, conversion from AGB to SG is an exciting option for revision surgery (5, 18, 19). Several clinical studies showed significant results regarding conversion from AGB to SG (20, 21). However, the evidence in the literature regarding the effectiveness of OS-SG and TS-SG after failed AGB is inconsistent (15, 22–24). Due to the lack of evidence and the heterogeneous number of patients included in some clinical trials, there are still no clear guidelines for conversion from AGB to SG at One-Step or Two-Step (25, 26).

The present study analyzed patients who underwent revision surgery One-Step or Two-Step SG after failed AGB between 2005 and 2019. In addition to outcome analysis after primary SG (*n* = 27,346), we analyzed data on revision surgery complications. With a total of 488 patients (320 patients with OS-SG and 168 patients with TS-SG), our study represents a large reported series of SG after failed AGB. All other bariatric surgical procedures were not included. Our study investigates whether One-Step or Two-Step is superior to revision surgery after failed AGB and compares the outcomes of these two procedures with the outcome after primary SG. The time between the first and second surgery was 6 weeks to 6 months for the Two-Step approach. The decision for One- or Two-Step procedures and SG depends on the surgeon's experience, risk factors, and comorbidities. Based on the results of our study, the One-Step procedure was performed more frequently than a Two-Step procedure. Our study did not investigate the reason for choosing the procedure, removal of the gastric band, and surgeon's experience in performing One- or Two-Step procedures.

In the present study, we did not investigate the reason for the different distribution of BMI, gender, and age between the three groups.

The percentage distribution of comorbidities was significantly higher in P-SG than in OS- and TS-SG (*p* < 0.001). The incidence of reflux, gonarthrosis, pulmonary comorbidities, arterial hypertension, sleep apnea, and degenerative skeletal disease was significantly different between the three groups (Table 2). However, this comparison was made before revision surgery in the OS- and TS-SG groups and after implantation of the AGB. The effect of P- and R-SG to improve comorbidities was not investigated in this study. Long-term follow-up is needed to study the effect of revision surgery compared with primary surgery.

Several clinical trials have compared the long-term and short-term outcomes of primary SG and revision SG (R-SG) (27–29). Chansaenroj et al. analyzed data from 275 patients after revision surgery of a failed AGB. Several factors were considered, including percentage excess weight loss at 10-year follow-up, revision surgery, and major complication. Fifty-three patients (19.3%) underwent revision surgery [26 single anastomosis (mini-)gastric bypass (R-LSAGB), 17 sleeve gastrectomy (R-LSG), 9 Roux-en-Y gastric bypass (R-LRYGB), and one other procedure]. After revision surgery, there was a significant mean excess weight loss (EWL%) of over 50% compared to 45% in the post-gastric banding group at the 10-year follow-up. In the conclusion of the study, the authors stated that revision surgery SG of failed AGB is safe and can be performed without increased complication rates (27). This is consistent with the results of our analysis. In general, our study showed that R-SG has a favorable outcome in terms of short-term outcomes in patients with obesity and failed AGB. However, we did not analyze the effects of R-SG on the change in BMI and %EWL. This should be done in other studies with long-term follow-up after revision surgery.

Based on a literature review, the most commonly documented intraoperative and post-operative complications after revision SG were an injury to the diaphragm, pneumothorax, pleural empyema, gastric perforation, injury to the spleen, and liver (16, 30). P-SG was associated with significantly lower complication rates compared with R-SG (19). In our study, the intraoperative complication rate was lower after P-SG than after R-SG (1.4% for P-SG vs. 3.8% for OS-SG and 3% for TS-SG; *p* < 0.001). Analysis of total general post-operative complications showed no significant difference between the three groups. However, the analysis of individual general post-operative complications showed a significantly higher incidence of pulmonary complications for OS-SG than for TS- and P-SG (*p* < 0.001). With a *p*-value above 0.05, there was no significant difference between the three groups regarding the incidence of other documented general post-operative complications.

Despite the benefits of revision surgery after failed AGB, many studies investigated the effect and safety of One-Step vs. Two-Step SG. Schneck et al. (31) analyzed data from 2,061 patients with One-Step and 1,296 patients with Two-Step revision surgery after failed AGB in a retrospective study. 11.7% of patients after OS-SG required an intensive care unit stay compared to 6.7% for TS-SG (*p* < 0.001). In addition, the complication rate was significantly higher in OS-SG than in TS-SG (15.9 vs. 12.7%; *p* = 0.009). Thus, the study suggests that TS-SG results in a significantly lower post-operative complication rate than OS-SG. Stroh et al. (15) compared the post-operative outcome of 137 patients after OS-SG and 37 patients after TS-SG as revision surgery after AGB failure. The post-operative staple line leakage rate was higher for OS-SG (4.4%) than for TS-SG (0%). A meta-analysis (32) of nine studies involving 809 patients after OS-SG and TS-SG compared the post-operative outcomes in both groups. The complication rate was significantly higher in OS-SG than in TS-SG. In addition, complication management was associated with higher costs (mean $806) after OS-SG than in the TS-SG group. The length of hospital stay was longer in the TS-SG group than in the OS-SG group, with no significant effect. The study suggests that TS-SG is associated with a lower complication rate and lower costs compared to OS-SG. However, some clinical studies showed similar results regarding the post-operative outcome of OS-SG and TS-SG (23). In our study, the total intraoperative complication rate distribution differed between the two groups (OS-SG and TS-SG). There was a significant difference in favor of TS-SG compared with OS-SG (3 vs. 3.8%; *p* < 0.001). Notably, there was a significant disadvantage between the two groups in gastric perforation in favor of TS-SG (0.9% for OS-SG and 0% for TS-SG; *p* < 0.001). However, the splenic injury was significantly higher in TS-SG (0.6% in OS-SG and 1.8% in TS-SG; *p* = 0.003). In terms of other intraoperative complications, there was no significant disadvantage between the two groups, with a *p*-value above 0.05 (Table 4). The total general post-operative complication rate was not significantly different between the two groups (*p* = 0.267). Regarding individual complications, the incidence of pulmonary complications was significantly higher in the OS-SG (3.1% OS-SG vs. 0% TS-SG; *p* < 0.001). There was no significant difference in the incidence of other documented complications between the two groups, with a *p*-value >0.05. The total rate of specific post-operative complications was insignificant in the two groups (6.3% in OS-SG and 3% in TS-SG; *p* = 0.68). In particular, there was a significant difference in staple line stenosis in favor of TS-SG (0.6% in OS-SG and 0% in TS-SG; *p* = 0.005) and the occurrence of sepsis (1.6% in OS-SG and 0% in TS-SG; *p* = 0.008). We found no significant difference in the incidence of other complications analyzed between the two groups (Table 5).

The operating time differed significantly among the three groups, with the least time in the P-SG group followed by OS-SG and TS-SG (74.8 min for the P- SG group vs. 113.8 min for the OS-SG vs. 162.3 min for the TS-SG group; *p* < 0.001). The difference in mean operating time between TS-SG and OS-SG may be explained because skin incisions, trocar placement, and skin sutures had to be performed twice in the TS-SG group compared with the OS-SG group.

Several clinical trials have examined post-operative mortality after bariatric surgery. According to the American College of Surgeons, one study showed an overall low mortality rate after bariatric surgery (33). Compared to other surgical procedures such as Roux-en-Y gastric bypass (RYGB), 30-day mortality was significantly lower after P-SG than after RYGB (0.09% after SG vs. 0.14% after RYGB; *p* = 0.01) (34). In our study, the mortality rate was not significantly different between the three groups (0.2% after P- SG vs. 0.3% after OS-SG vs. 0% after TS-SG; *p* = 0.727).

## Conclusions

A failed AGB is best treated with conversion to another bariatric procedure. The present study found that conversion to SG can be performed at One-Step or Two-Step for failed AGB with nearly comparable post-operative outcomes. In the short term, primary surgery (P-SG) appeared to have the lowest complication rates in all groups compared with One-Step or Two-Step revision surgery. However, OS-SG had significantly higher pulmonary comorbidities compared to TS- and P-SG. Regarding the surgery-related post-operative complication rate, OS-SG showed an overall higher complication rate than TS-SG and P-SG. The mean operating time was significantly lower in OS-SG than in TS-SG but higher than in P-SG. In terms of 30-day mortality, there was no significant disadvantage in the three groups.

SG as a redo procedure after failed AGB is safe and beneficial. OS-SG and TS-SG provide an acceptable outcome in terms of intraoperative and post-operative complication rates. Despite the advantages of both procedures, we cannot recommend any procedure in our study. Both procedures (OS-SG and TS-SG) are safe and suitable for revision surgery. However, it should be noted that proper patient selection is essential to avoid such possible adverse complications. The indication should be individualized and dependent on the intraoperative findings and the general condition of the patient. Concerning long-term results, further studies with higher methodological quality are required.

## Summary

The last few decades have brought enormous medical development in many areas, including bariatric and metabolic surgery. Despite this development, there are still uncertainties as to which surgical method represents the best outcome for the patient. Furthermore, if a one surgical method fails, alternative therapy methods should be used to achieve an adequate outcome for the patients with obesity.

One of the best-known bariatric surgery methods is the gastric band. Despite the many advantages of this method, clinical studies have shown that it has an insufficient effect on weight reduction and the treatment of obesity-related diseases. In this case, other surgical methods, such as the formation of a sleeve gastrectomy, should be applied. However, the question is how to perform such a surgery. Is a one-stage sleeve gastrectomy (simultaneous removal of the gastric band and formation of sleeve gastrectomy) or a two-stage surgery (removal of the gastric band in the first surgery and formation of the sleeve gastrectomy in the second surgery) more beneficial for patients after insufficient gastric band implantation? This has been investigated in our study. As a conclusion, we found that the two methods, compared to the formation of the sleeve gastrectomy as primary procedure, are safe and can be performed with fewer complications. The two procedures differed minimally on individual aspects, but the general end result was almost identical in the two groups.

## Data Availability Statement

The raw data supporting the conclusions of this article will be made available by the authors, without undue reservation.

## Ethics Statement

The studies involving human participants were reviewed and approved by Ethikkomitee der Otto-von Guericke Universität Magdeburg. The patients/participants provided their written informed consent to participate in this study.

## Author Contributions

OT study concept, literature review, manuscript writing, and revision of manuscript. JD literature search and manuscript writing. MH preparation of data for statistical analysis and approval of statistical results and outcomes. FK and CS study concept, preparation of data for statistical analysis, revision of manuscript, and final approval of the manuscript. All authors contributed to the article and approved the submitted version.

## Conflict of Interest

MH was employed by the company StatConsult GmbH, Magdeburg. The remaining authors declare that the research was conducted in the absence of any commercial or financial relationships that could be construed as a potential conflict of interest.

## Publisher's Note

All claims expressed in this article are solely those of the authors and do not necessarily represent those of their affiliated organizations, or those of the publisher, the editors and the reviewers. Any product that may be evaluated in this article, or claim that may be made by its manufacturer, is not guaranteed or endorsed by the publisher.

## Abbreviations

AGB: Adjustable Gastric Banding
BMI: Body mass index
GBSR: German Bariatric Surgery Registry
OS: One-Step
OS-SG: One-Step Sleeve Gastrectomy
P-SG: Primary Sleeve Gastrectomy
R-SG: Revision Sleeve Gastrectomy
SG: Sleeve Gastrectomy
SPC: Specific post-operative complications
TS: Two-Step
TS-SG: Two-Step Sleeve Gastrectomy
%TWL: %total weight loss.
